# Numerical Simulations of Carbon-Fibre Impregnation with a Polymer as an Anisotropic Permeability Medium

**DOI:** 10.3390/ma16206627

**Published:** 2023-10-10

**Authors:** Daniel Gomes, Luís Amorim, Raquel M. Santos, Nelson D. Gonçalves

**Affiliations:** 1 Institute of Science and Innovation in Mechanical and Industrial Engineering (INEGI), Rua Dr. Roberto Frias, 400, 4200-465 Porto, Portugal; dgomes@inegi.up.pt (D.G.); luis.amorim.gm@gmail.com (L.A.); rmsantos@inegi.up.pt (R.M.S.); 2 LAETA—Associated Laboratory of Energy, Transports and Aerospace, 4200-265 Porto, Portugal

**Keywords:** carbon-fibre-reinforced polymers, anisotropic permeability, porous medium, computational fluid dynamics

## Abstract

The impregnation process of carbon fibres with polymers is challenging to model due to the system’s complexity, particularly concerning the following aspects: the complex rheology of the polymeric matrices and the presence of solid, continuous fibres, both with anisotropic properties, and the interaction between solid and fluid, which can change the displacement of fibres into a cyclic dependence. In this work, an interesting approach was considered by setting the fibres as a porous medium whose properties were calculated with microscale/macroscale cycle modelling. In the microscale modelling stage, two main assumptions can be made: (i) a homogeneous distribution with a representative cell or (ii) a stochastic distribution of fibres. The solution to the abovementioned flow and fibre distribution problem can severely differ with only a slight change in a single parameter for a given set of processing parameters. Therefore, the influence of some of them during the fibre impregnation process was evaluated, allowing a shortcut for the polymer through a gap between fibres and the bottom wall of the extrusion die. The range of investigated values regarding the gap enables one to cover good impregnation conditions up to the occurrence of the shortcut and consequent poor impregnation quality. These studies were performed with numerical simulations with circa 126,000 degrees of freedom, considering the discretisation mesh elements and the unknowns (pressure and two velocity components).

## 1. Introduction

Carbon-fibre-reinforced polymer (CFRP) composites have been replacing traditional materials in a wide range of advanced applications, from aerospace, defence and aeronautics to sports, renewable energies and civil engineering, among others [1,2,3,4,5,6,7,8,9,10]. The reasons for the increasing demand for composites are due to not only their remarkable in-plane mechanical properties (stiffness and strength), fatigue and corrosion resistance, durability and light weight but also their high degree of freedom when designing and engineering complex structures according to the final application [11,12,13]. The correct selection and conjugation of constituent materials (matrix/reinforcement) of CFRP can significantly influence the final part’s processability, performance, sustainability and cost. High-performance reinforcements (e.g., carbon fibres) can substantially increase the part’s final price, while conventional matrices (e.g., polyester and epoxy resins) can facilitate processing, although they will compromise recyclability and repairability [14,15]. Last but not least, the possibility to nano-reinforce CFRP constituents can contribute to additional improvements in out-of-plane mechanical, thermal and electrical properties and also address new multifunctional requirements, such as self-healing, structural health monitoring (SHM) and electromagnetic interference (EMI) shielding, among others [16,17,18,19].

Traditionally, thermoset (TS) resins have been used as the preferential matrix system of CFRPs. Before curing reactions, a TS presents low viscosity, which facilitates resin impregnation on the reinforcements, usually driven by pulling the fibre through a set of cylindrical pins and a resin bath. This low-pressure process usually results in poor or incomplete fibre impregnation on pre-impregnated materials and, consequently, unpleasant defects in the developed material, which may compromise the overall properties of the CFRP composites. However, achieving pre-forms with a high volume fraction of carbon fibre is possible if specific attention is paid to processing parameters and material properties such as fibre pre-tension, fibre permeability, pressure build-up, residence time, pulling tension and polymer viscosity during pin-assisted resin infiltration [20]. Throughout the curing process, a three-dimensional network of irreversible cross-linked molecules is formed, conferring to the system volumetric stability and relatively high stiffness (around 3GPa for a typical epoxy), even at moderately high temperatures (around 100 ${}^{\circ }$C) [21]. Nevertheless, the production of high-quality composite parts based on TS matrices usually requires long curing and post-curing stages in an autoclave under high pressure and temperature to minimise voids and dry fibres as well as to ensure an appropriate and full matrix cure. This time- and energy-consuming process, associated with manual layup and long labour time, leads to a low-volume production rate and expensive composite parts [22,23].

Accordingly, thermoplastic (TP)-based composites are a promising alternative to replace their TS counterparts for highly demanding applications. At the molecular structural level, TP polymers are composed of long, branchy molecules, typically linked by weak secondary intermolecular bonds and forces, such as hydrogen or van der Waals bonds. When heated, those bonds can be temporally broken, allowing molecular rearrangements, thus enabling welding, repeatability and recyclability [24,25]. Additionally, most of them present higher mechanical properties under impact and shear loading conditions when compared with TSs [26,27]. Taking advantage of the weldability features of TP matrices and recent automated placement manufacturing technologies, including automated fibre placement (AFP) and automated tape laying (ATL), TP composites can be consolidated quickly in complex geometries in situ by applying pressure and temperature, reducing the production time, leftovers and costs associated with high-energy-consuming equipment, such as autoclaves [22,28].

Nevertheless, compared to TS resins, the long molecular chains significantly increase the viscosity of TP polymers at the melting stage, preventing good fibre impregnation. This may lead to dry fibre spots in the composite and, consequently, inefficient loading transfers from the matrix to reinforcements. This drawback can be overcome by the development of continuous TP pre-impregnated intermediate products in dry (e.g., towpregs and commingled yarns) [29,30] or pre-consolidated (e.g., unidirectional tapes) [11,31] forms. Dry pre-impregnated TP composites are economically attractive since they can be produced continuously with low-pressure and low-temperature processes, although damages and an uneven distribution of reinforcement fibres may arise from the poor control of impregnation variables [32,33]. On the other hand, pre-consolidated TP composites can be produced through solvent or hot-melt impregnation processes. The former consists of dipping fibres continuously in a bath of TP polymer mixed with a solvent to reduce their viscosity. However, the chemical affinity between solvent and polymer, and the usage of very hazardous solvents for high-performance polymers and their residues on the final pre-impregnated tape, as well as environmental considerations related to this process, are some of the challenges of this technology [31,34]. The latter, hot-melt impregnation, consists of using high temperatures and pressures to melt or soften the polymer and impregnate fibres in a continuous co-extrusion process. Typically, spread reinforcement fibres enter an extruder die designed to soften the polymer at the correct temperature, pressure and residence time to ensure fibre impregnation. The final pre-impregnated composite, in tape form, is wound on a spool afterwards [11]. This process allows high production rates of thin unidirectional pre-impregnated TP tapes with high-volume fibre content and fine quality. Nevertheless, process variables—namely, fibre spreading, tension and feeding rate; melt temperature and pressure into the die; as well as die design—are key aspects of the process that should be carefully controlled and considered to yield high-quality tapes.

Accordingly, this work aims to study and understand the role of hot-melt process variables on a new die geometry and how its design can be improved to ensure efficient impregnation. The influence of solid, long fibres on the flow can be modelled through a porous media, where the flow rate and pressure drop can be predicted by Darcy’s law [35], where the permeability (the ability for fluids to flow through) is evaluated by geometric properties, such as the shape of fibres, their stochastic distribution, tortuosity of the medium [36,37,38,39,40] or even their surface properties. The isotropic permeability can be generalised to the anisotropic case [41], considering the orientation of the fibres: (i) flow across (perpendicular to) fibres and (ii) flow parallel to fibres. In addition to the analytical solution and numerical simulation, experimental data [42] can be analysed to estimate model parameters.

The present work will feature a section of an in-development extrusion die, which co-extrudes polymer and carbon fibres aiming to produce a tape of carbon fibres. In Section 2, the relevant modelling equations to the process are presented. In Section 3, the geometry of the problem and the material properties are described, as well as the discretisation details and numerical codes employed to solve the equations. Before the analysis of the results, some considerations regarding the expected dependence of the flow across the fibres versus the path between fibres and the extrusion die wall are presented, and the expected displacement of fibres due to the flow is also analysed (Section 4). The results are described and analysed in Section 5, and a die geometry is proposed, considering a range of realistic processing parameters. Finally, the main conclusions are summarised in Section 6.

## 2. Governing Equations

Aiming at modelling the flow throughout continuous fibres, an anisotropic medium similar to a porous medium is considered, where the pressure drop is modelled by Darcy’s Law [35], according to Equation (1):

(1) $$\frac{\partial p}{\partial x_{i}}=-\frac{\mu }{K_{i}}u_{i},$$

where *p* is the pressure, $x_{i}$ is a direction on the porous medium, $u_{i}$ is the fluid i−th velocity component, $K_{i}$ is the porous medium permeability in the same direction and μ is the fluid dynamic viscosity. Since there are two main flow directions, namely, parallel (‖) and perpendicular (⊥) to the fibres, a generalisation of each one was considered, taking into account their corresponding permeabilities.

**Figure 1:**
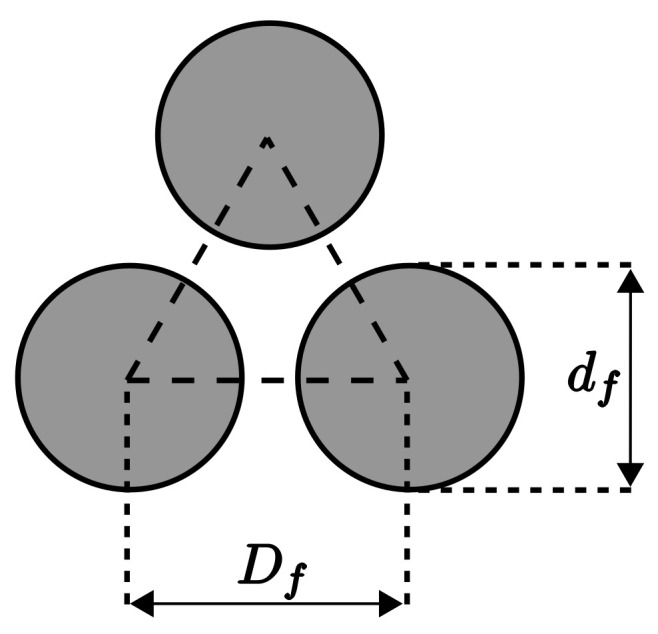
Fibres’ displacement with their diameter $\left(d_{f}\right)$ and distance between centres $\left(D_{f}\right)$.

The permeability modelling strongly depends on the cross-section fibre arrangement, typically performed by empirical evaluations from stochastic distributions obtained using laboratory measurements or numerical simulations. Another possible approach is the assumption of repetitive distribution and theoretical simplifications, such as those performed, e.g., by Gebart [41]. This last approach was used in this work with the hexagonal arrangement of the fibres, where each fibre is surrounded by six other equally spaced fibres. Despite the rough simplification, this will allow the study of other parameters, including fibre displacement and extrusion die geometry, which is the main goal of this work. The porous medium is schematised in Figure 1 considering its main geometric dimensions, namely, the fibres’ nominal diameter $d_{f}$ and the distance between their geometric centres $D_{f}$.

Gebart [41] deduced a permeability approach of a porous media defined by fibres, with permeability in the fibres’ direction $\left(K_{\|}\right)$ or in the perpendicular direction $\left(K_{⊥}\right)$, given by Equations (2) and (3), respectively:

(2) $$K_{\|}=\frac{2d_{f}^{2}}{53}\frac{{\left(1-V_{f}\right)}^{3}}{V_{f}^{2}},$$

(3) $$K_{⊥}=\frac{4d_{f}^{2}}{9\pi \sqrt{6}}{\left(\sqrt{\frac{V_{fmax}}{V_{f}}}-1\right)}^{5/2},$$

where $V_{f}$ is the fibre volume fraction and $V_{fmax}$ is the maximum value, i.e., the value of $V_{f}$ when fibres touch each other $\left(d_{f}=D_{f}\right)$. Assuming the triangle in Figure 1, $V_{f}$ can be calculated by

(4) $$V_{f}=\frac{3\frac{60}{360}\frac{\pi d_{f}^{2}}{4}}{\frac{1}{2}D_{f}\frac{\sqrt{3}}{2}D_{f}}=\frac{\pi }{2\sqrt{3}}{\left(\frac{d_{f}}{D_{f}}\right)}^{2}.$$

From this last expression, its maximum value is derived from $V_{fmax}=\frac{\pi }{2\sqrt{3}}$ and $\frac{V_{f}}{V_{fmax}}={\left(\frac{d_{f}}{D_{f}}\right)}^{2}$.

Assuming that a certain number of fibres $N_{f}$ with diameter $d_{f}$ are placed on a rectangle of width *W* and height $H_{f}$, the volume fraction is $V_{f}=\frac{N_{f}\pi d_{f}^{2}}{4WH_{f}}$. Thereby, $D_{f}$ can be evaluated by $D_{f}=\frac{d_{f}}{\sqrt{V_{f}/V_{f}max}}$ and the minimum value to $H_{f}$, $H_{fmin}$, can be obtained from $V_{f}=V_{fmax}$:

(5) $$H_{fmin}=\frac{N_{f}d_{f}^{2}\sqrt{3}}{2W}.$$

To model the flow of an incompressible and generalised Newtonian fluid under isothermal conditions, the Navier–Stokes equations were employed, namely, the mass conservation equation

(6) $$\frac{\partial \left(\rho u\right)}{\partial t}+\nabla \;\cdot \;\left(\rho u\right)=0,$$

and the momentum conservation equation

(7) $$\frac{\partial \left(\rho u\right)}{\partial t}+\nabla \;\cdot \;\left(\rho uu\right)=\nabla \;\cdot \;\tau -\nabla p+S_{u},$$

where ρ is the fluid density, u is the fluid velocity, τ is the stress tensor, $\tau_{i,j}=\eta \left(\dot{\gamma }\right)\left(\frac{\partial u_{i}}{\partial x_{j}}+\frac{\partial u_{j}}{\partial x_{i}}\right)$, $\dot{\gamma }$ is the shear rate, *p* is the pressure and $S_{u}$ is the source term.

In this work, the Bird–Carreau viscosity [43,44] model was considered to model the viscosity of the generalised Newtonian fluid, accordingly to

(8) $$\eta \left(\dot{\gamma }\right)=\eta_{\infty }+\frac{\eta_{0}-\eta_{\infty }}{{\left(1+{\left(\lambda \dot{\gamma }\right)}^{2}\right)}^{\frac{1-n}{2}}},$$

where $\eta_{0}$ is the viscosity at zero shear rate, $\eta_{\infty }$ is the viscosity at infinite shear rate, λ is the characteristic time and *n* is the power index.

Considering Equation (1) and the fibres’ velocity in direction *i*, $u_{fi}$, and fluid velocity in the same direction, $u_{i}$, the source term added to model the pressure drop due to the resistance for the fluid to pass through the fibres is

(9) $$S_{i}=\frac{\eta }{K_{i}}\left(u_{fi}-u_{i}\right),$$

leading to $S_{\|}=\frac{\eta }{K_{\|}}\left(u_{f}-u\;\cdot \;e_{\|}\right)$ and $S_{⊥}=\frac{\eta }{K_{⊥}}\left(0-u\;\cdot \;e_{⊥}\right)$, where $e_{i}$ is the unitary vector in the direction *i*. Notice that the velocity used on these source terms results from the difference between the fibre and fluid velocities since it is equivalent to the porous medium, i.e., the fibres are moving.

The source term of the momentum conservation equations $\left(S_{u}=S_{u}e_{x}+S_{v}e_{y}\right)$ is obtained by projecting the parallel and perpendicular components in the respective directions, i.e., $S_{u}=S_{\|}e_{\|}\;\cdot \;e_{x}+S_{⊥}e_{⊥}\;\cdot \;e_{x}$ and $S_{v}=S_{\|}e_{\|}\;\cdot \;e_{y}+S_{⊥}e_{⊥}\;\cdot \;e_{y}$, where $e_{i}$ is the unitary vector on direction *i*.

### Numerical Simulations

The software Gmsh (https://onlinelibrary.wiley.com/doi/abs/10.1002/nme.2579 (accessed on 7 September 2023)) [45] was used for the geometry definition and its discretisation. In addition, OpenFOAM 7 was employed to solve the previously mentioned governing equations. This numerical code is able to deal with unstructured meshes, applying the Finite Volume Method (FVM) by integration on space and time, leading to a system of equations whose solution is the velocity components and pressure in each element. Although it is a steady state problem, a time evolution was considered to deal with the instabilities of the converging algorithm. The velocity pressure coupling was solved with the SIMPLE algorithm (simpleFoam) using standard method parameters defined in OpenFOAM. The graphical analysis of results was achieved with ParaView (version 5.6.0, 64-bit), and the data analysis was performed with Python (version 3.9, 64-bit) codes.

## 3. Study Case

### 3.1. Geometry

**Figure 2:**
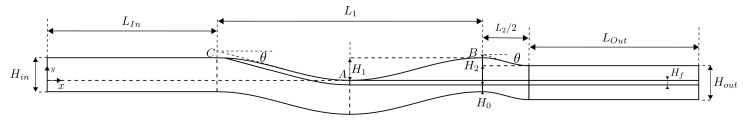
Fluid domain with geometry parameters and fibres location.

The geometry section considered in this work (Figure 2) comprises a straight entry section, with two sequential waves and a straight end section. In this geometry, a set of 60,000 fibres, with nominal diameter $d_{f}=7\;\mu m$, enters through the top of the extrusion die on point C and, due to the sinusoidal shape of the domain walls, the polymer is forced to pass through fibres, mainly between point A and point B.

When the polymer passes through the fibres, there is a pressure differential between the lower and upper part of fibres that will be correlated with the mass flow through the fibres and the permeability of fibres. This pressure differential forces the fibres upwards, imposing a vertical displacement on the fibres and varying the gap’s height between the fibres and the bottom die wall on point B. The following can be inferred:

- The gap between the fibres and the extrusion die wall enables a direct passage—a shortcut—of the fluid. The gap’s height will then influence the fluid pressure drop at point B, and the mass flow rate through the gap and through the fibres.
- The fibre displacement in the vertical direction $\delta_{y}$ will be defined by the pressure differential through the fibres and the tension applied to the fibres by the pulling system.

**Figure 3:**
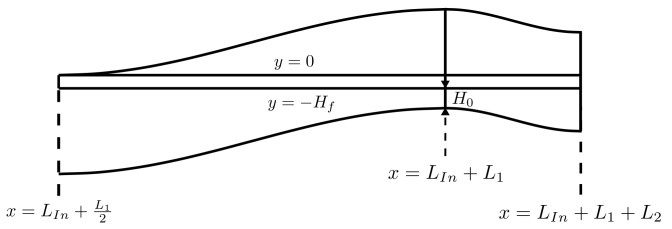
Closer view to fluid domain between points A and B with reference lines.

To analyse the fibre displacement $\delta_{y}$ in relation to the pressure differential through the fibres, a coupled simulation of Computational Fluid Dynamics (CFD) and Finite Element Analysis (FEA) is needed. A simplification was defined, where by maintaining a fixed die geometry and a constant fibre position, $\delta_{y}=0$, the gap dimension $H_{0}$ varies with the thickness of the fibres layer $H_{f}$, (see Figure 2 and Figure 3). The lower and higher compression of the fibres is defined by the fibres layer thickness $H_{f}$, which influences the permeability, allowing the analysis of the flow through the fibres at different permeability values.

Based on the definition, the porous medium, i.e., the fibres, will have a constant shape ($\delta_{y}=0$) but the gap’s dimension will vary to evaluate its influence on the flow. Later, an analysis of $\delta_{y}$ evolution with the pressure drop through the fibres will be implemented to define the optimum geometry for the die in correlation with the desired gap and a range of tensions applied to the fibres.

In Figure 2, the first wave shape with length $L_{1}$ and height $H_{1}$ at the fibres entrance can be seen. A second (half) wave is defined with length $L_{2}/2$ and height $H_{2}$. These curves were set with a sinusoidal shape, accordingly with

(10) $$y=y_{i}+\frac{H_{i}}{2}\left(-1+cos\left(2\pi \frac{x-x_{i}}{L_{i}}\right)\right),$$

where $x_{i}$ and $y_{i}$ are the coordinates of the starting point. Both lengths $L_{1}$ and $L_{2}$ were set to obtain a maximum slope of θ=15°, i.e., slope ${\left[\frac{dy}{dx}\right]}_{x=L_{i}/4}=-tan\left(15{}^{\circ}\right)=-\left(2-\sqrt{3}\right)=m$, leading to

(11) $$L_{i}=H_{i}\pi \left(2+\sqrt{3}\right)=\frac{H_{i}\pi }{-m}.$$

Specifying that the fibres should be equally spaced from the top and bottom walls at the output, one can obtain $H_{1}-H_{2}=H_{0}+H_{2}$, leading to the definition of $H_{2}$ from the values of $H_{1}$ and $H_{0}$, i.e., $H_{2}=\frac{H_{1}-H_{0}}{2}$. It can be seen that $H_{in}=H_{0}+H_{f}+H_{1}$ and $H_{out}=\left(H_{0}+H_{2}\right)+H_{f}+\left(H_{1}-H_{2}\right)$; therefore, $H_{In}=H_{Out}$. In this work, we considered the height of $H_{In}=H_{Out}=1.5\;\mathrm{mm}$. The height of the first wave was set to $H_{1}=1\;\mathrm{mm}$, leading to $H_{f}+H_{0}=0.5$. The lengths $L_{In}$ and $L_{Out}$ are defined as five times the height of the channel, i.e., $L_{In}=L_{Out}=7.5\;\mathrm{mm}$. From the $H_{1}$ value and using Equation (11), the value $L_{1}\approx 11.7\;\mathrm{mm}$ is derived. Since $H_{2}$ is a function of the gap $H_{0}$, and this depends on $H_{f}$, the length $L_{2}$ also varies with $H_{f}$. Using Equation (5), one can obtain the minimum thickness of the fibres layer $H_{fmin}\approx 0.101\;\mathrm{mm}$. Several numerical simulations can be performed by varying the values of $H_{f}$ and $H_{0}$, allowing the analysis of each parameter’s influence on the polymer flow through the die geometry.

### 3.2. Mesh

**Figure 4:**
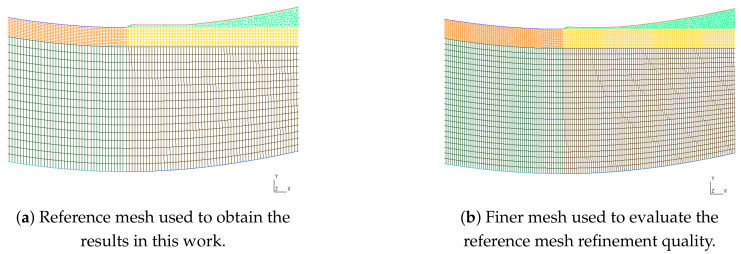
Mesh comparison near the fibres’ detachment from the upper wall.

The mesh was defined in a structured way all over the domain, except where the fibres detach from the top wall (see Figure 4). Downstream from this point, due to the generation of stretched elements, triangles were used, and the minimum height of one mesh element between the fibres and the top wall was assured to avoid convergence problems that would otherwise occur.

As a first attempt, the geometry with the fibres’ thickness layer $H_{f}=0.2\;\mathrm{mm}$, the horizontal and vertical lines were discretised with elements of 0.03mm in length and elements along the fibres layer were considered with 0.015mm. This reference mesh has around 42,000 cells (34,381 hexahedrons and 7941 prisms). A finer mesh was defined, dividing the previous elements’ length by $\sqrt{2}$, leading to a mesh around two times the number of elements.

**Figure 5:**
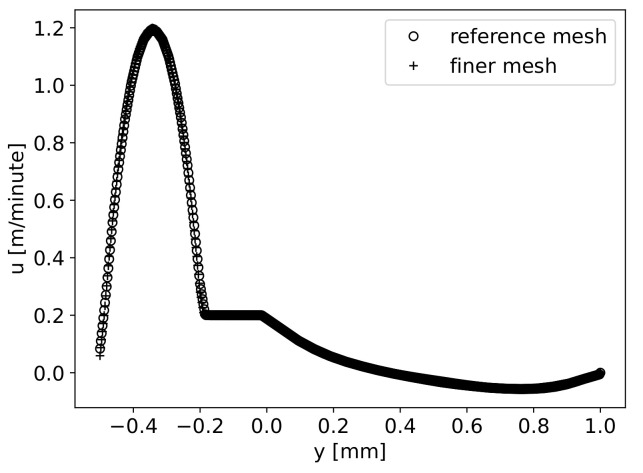
Horizontal velocity component along $x=L_{In}+L_{1}$: comparison of results obtained by two meshes.

Aiming to evaluate the mesh influence on the final result, the same problem was solved with both meshes (with the boundary conditions and material properties described below and $H_{f}=0.2\;\mathrm{mm}$). The horizontal component of velocity was compared along the vertical line at $x=L_{In}+L_{1}$, i.e., after the first wave. It can be seen that similar results were obtained with both meshes (see Figure 5). The results presented hereafter were obtained with the reference mesh.

### 3.3. Boundary Conditions

For the fibres’ velocity magnitude $U_{f}$, a common velocity was considered in the extrusion process, 200mm/min. At the inlet, the polymer was set with the corresponding average velocity, with a parabolic profile. At the outlet, the atmospheric pressure was set, i.e., 0.1MPa. A no-slip condition was defined all over the internal walls.

The region with fibres was considered part of the fluid with a source term (Equation (9)) modelling the fibres’ influence on the flow.

### 3.4. Rheological Parameters

The polymer considered in this study was polyphenylene sulphide (PPS), Xytron^TM^ U3020E, from DSM, with density $\rho =1290\;\mathrm{kg}\;\cdot \;m^{-3}$. To model the viscosity, a set of viscosity–shear rate data [46] were fitted with the Bird–Carreau equation (Equation (8)), leading to the following parameters: zero shear rate viscosity $\eta_{0}=202.9\;\mathrm{Pa}\;\cdot \;s$, infinite shear rate viscosity $\eta_{\infty }=0\;\mathrm{Pa}\;\cdot \;s$, characteristic time λ=0.00344s and power index n=2.

## 4. Preliminary Considerations

### 4.1. Simplified Model

Aiming at understanding the flow balance through the gap and through the fibres, a simpler situation can be considered with two dependent possibilities for the polymer to flow through: the fibres layer with thickness $H_{f}$ and permeability $K_{⊥}$, and a gap with pressure drop modelled as a flow between parallel plates along $L_{x}$. $L_{x}$ is an arbitrary value that can later be adjusted to the simulation results to better fit this model with the analysed flow. Since $L_{x}$ is correlated with $H_{0}$, it can be defined $L_{x}=a\;H_{0}$. In a balanced situation, the two pressure drops should be equal and the inlet flow rate, $q_{In}=H_{In}u_{In}$, equals the sum of flow through the fibres, $q_{v}=\frac{L_{1}}{2}v$, plus the flow through the gap, $q_{u}=H_{0}u$. These conditions are summarised in the following system of equations:

(12) $$\left\{\begin{array}{cc} \; & \frac{\Delta p_{x}}{L_{x}}=-\frac{12}{H_{0}^{2}}\mu u\\ \; & \frac{\Delta p_{y}}{H_{f}}=-\frac{\mu }{K_{⊥}}v\\ \; & \Delta p_{x}=\Delta p_{y}\\ \; & H_{In}u_{In}=\frac{L_{1}}{2}v+H_{0}u \end{array}\right.,$$

whose solution is a function of $H_{f}$ and $L_{x}$:

(13) $$\left\{\begin{array}{cc} \; & u=\frac{H_{in}u_{in}}{\frac{6L_{1}L_{x}K_{⊥}}{H_{0}^{2}H_{f}}+H_{0}}\\ \; & v=\frac{12K_{⊥}L_{x}}{H_{0}^{2}H_{f}}u \end{array}\right..$$

**Figure 6:**
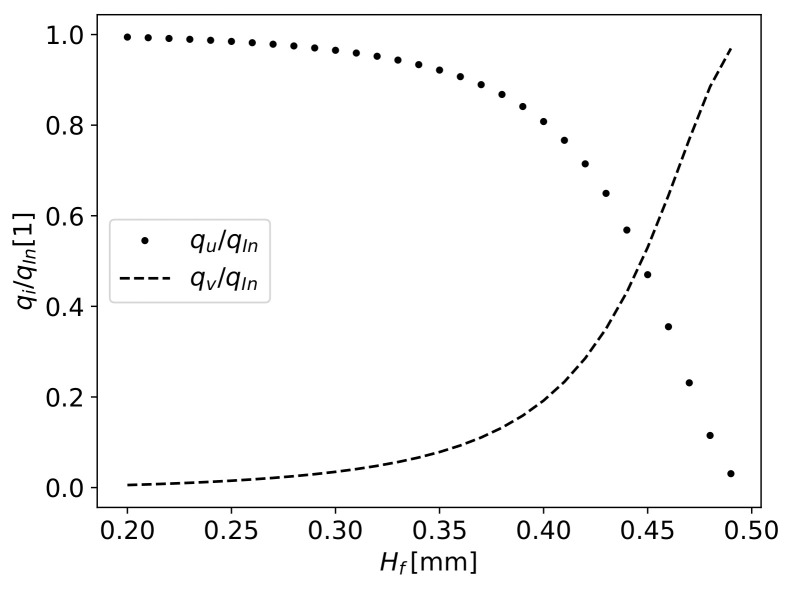
Volume flow rate for equilibrium pressure drops through fibres and through gap (assuming $5H_{0}$ as its length).

To analyse the flow distribution through the gap and the fibres prior to the simulations, $a=L_{x}/H_{0}$ was defined with an arbitrary value of 5 (see Figure 6). In this plot, the flow rates are normalised with the inlet value $q_{In}$.

It can be observed that the polymer fully flows through the gap until the value of $H_{f}=0.3\;\mathrm{mm}$. After this point, aiming to decrease $H_{0}$ and consequently increase Δp in the gap, the polymer flow through the fibres increases exponentially. The definition of a narrow working zone for correct fibre impregnation at the $H_{f}$ value of 0.4 means only 20% of the polymer flows through the fibres, and when $H_{f}=0.5\;\mathrm{mm}$, the whole polymer flows through the fibres.

By varying values of *a*, the flow distribution presents the same shape as the one in Figure 6 with horizontal displacement of the intersection point. Since *a* models the length of the gap, its increase leads to the diminishing of the flow through the gap. The fast shift of flow rates among the two ways (gap and through fibres) still occurs for other values of *a*. Numerical results can be used to obtain data that enable the definition of this shape parameter.

Despite simplification, this analysis allows us to predict the flow balance dependent on $H_{f}$ and $H_{0}$. Therefore, it is expected to obtain faster changes in the results for lower $H_{0}$ values, and more numerical simulations must be considered near these conditions.

As will be seen during the analysis, the obtained flow distribution with numerical simulations has a similar behaviour to the result presented here.

### 4.2. Fibre Displacement

As previously stated in this work, for the simulation of the flow through the die, it was considered that the fibres keep their position regardless of the pressure gradients created in the fibre by the polymer flow. This approximation allows a great simplification of the model and will produce accurate results for a fibre displacement $\delta_{y}$, much smaller than the significant length of the fibre $L_{1}$, presented in Figure 2.

Nevertheless, $\delta_{y}$ will directly influence $H_{0}$, therefore influencing the polymer flow, imposing the need to correctly correlate $\delta_{y}$ with the pressure gradients in the fibre for a given flow.

The fibres’ vertical displacement is mainly dependent on the pressure drop through the fibres between point A and point B (see Figure 2), and the applied pulling force. One can define the pressure differential considering the polymer flow through the fibres in the vertical direction defined by Darcy’s Law, dependent on the fibres’ layer thickness $H_{f}$, and the corresponding permeability $K_{⊥}$ (Equation (3)), dependent on $H_{f}$,

(14) $$\begin{array}{c} \Delta p_{y}=-\frac{\mu H_{f}}{K_{⊥}}v, \end{array}$$

where μ is the polymer shear viscosity and ν is the polymer velocity through the fibres, which can be determined in relation to the polymer volume flow through the fibres $q_{f}$ by

(15) $$\begin{array}{c} v=\frac{2q_{f}}{L_{1}W}, \end{array}$$

where *W* is the extrusion die width $\left(25\;\mathrm{mm}\right)$.

The volume flow through the fibres $q_{f}$ to completely fill the spaces between the fibres can be defined, dependent on $H_{f}$, by

(16) $$\begin{array}{c} q_{f}=\left(H_{f}W-N_{f}\frac{d_{f}^{2}}{4}\pi \right)u_{f}=\left(1-V_{f}\right)H_{f}Wu_{f}=q_{space}, \end{array}$$

where $u_{f}$ is the fibre velocity of $200\;\mathrm{mm}\;\cdot \;\min^{-1}$.

**Figure 7:**
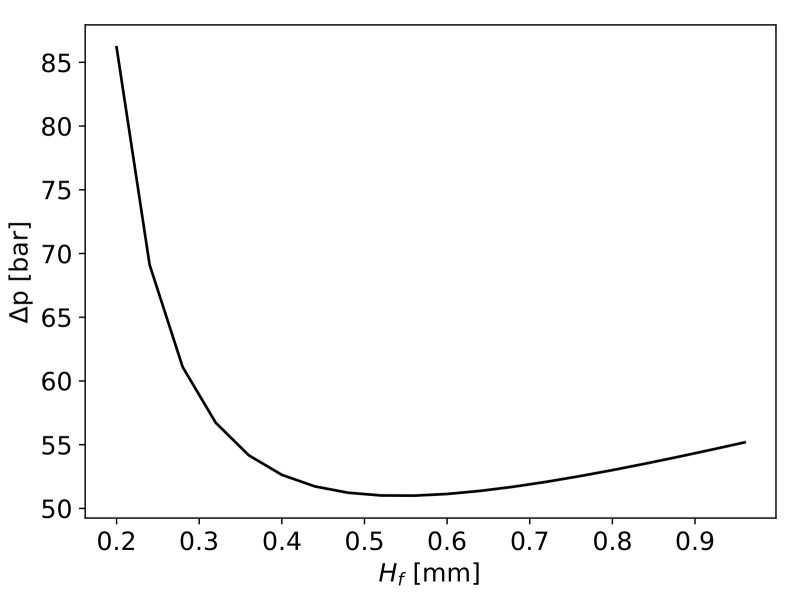
Pressure drop across fibres in correlation with the fibres’ height $H_{f}$.

The pressure drop in correlation with the height of the fibres layer $H_{f}$ is plotted in Figure 7.

The pressure drop through the fibres needed to fully impregnate the fibres is determined to be 86.2bar for an $H_{f}$ of 0.2mm and will exponentially decrease until an $H_{f}$ value of 0.56mm, where it is computed as 51bar. After this point, the pressure will increase at an almost steady rate of 11.7bar per mm of $H_{f}$.

**Figure 8:**
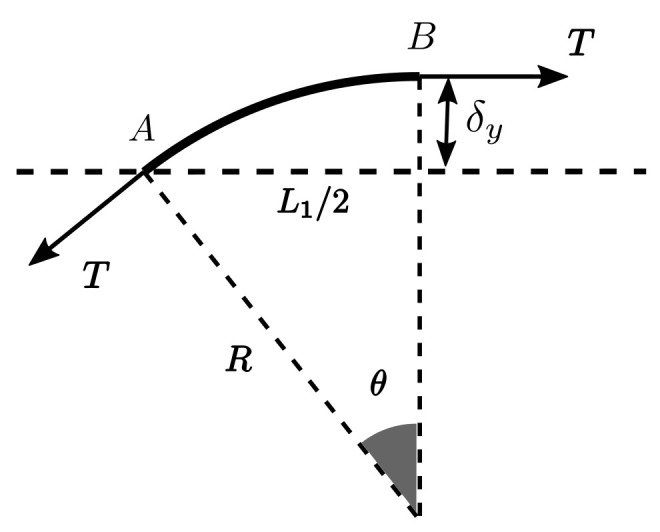
Considered fibre geometry between points A and B, and considered dimensions.

To analyse the fibre displacement and the applied tension, it was considered that the deformation of the fibres promoted by the pressure differential can be approximated by an arc of circumference between point A and point B, with radius R and angle θ (see Figure 8).

By the equilibrium of forces in the vertical direction, considering the polymer pressure differential Δp and the pulling force $F_{p}$ applied to the fibres, it is possible to correlate these two unknowns by

(17) $$\begin{array}{cc} \; & \Delta pW\frac{L_{1}}{2}=2F_{p}sin\left(\frac{\theta }{2}\right)\\ \Leftrightarrow & F_{p}=\frac{\Delta pWL_{1}}{4sin(\frac{\theta }{2})}, \end{array}$$

where $L_{1}/2$ is the length of the section between points A and B.

Using geometric relations (Figure 8), it is also possible to define the fibres’ vertical displacement $\delta_{y}$ as a function of θ with

(18) $$\begin{array}{cc} \; & R=Rcos\left(\theta \right)+\delta_{y}\\ \Leftrightarrow & \delta_{y}=R\left(1-cos\left(\theta \right)\right), \end{array}$$

and

(19) $$\begin{array}{cc} \; & sin\left(\theta \right)=\frac{L_{1}/2}{R}\\ \Leftrightarrow & R=\frac{L_{1}/2}{sin\left(\theta \right)}. \end{array}$$

**Figure 9:**
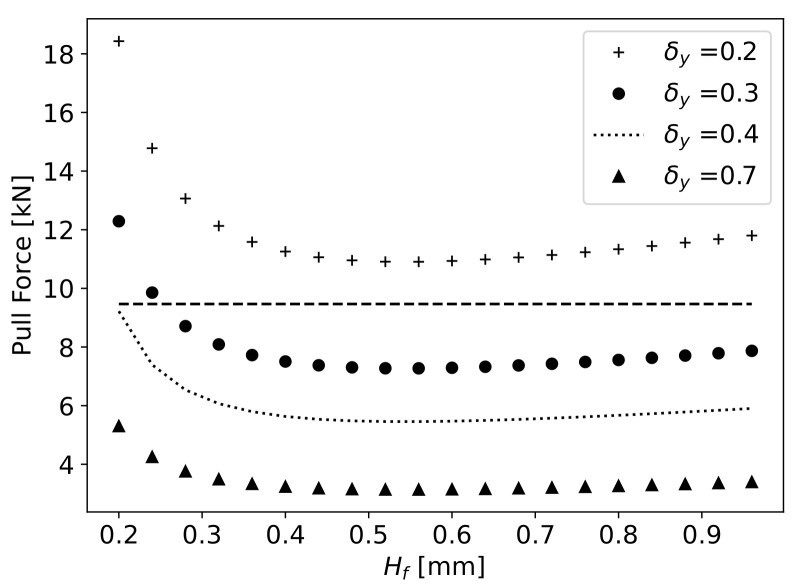
Pulling force applied to the fibres in relation to $H_{f}$ with varying $\delta_{y}$.

Considering the tensile strength of carbon fibres of 4100MPa [47], and the cross-sectional area of the 60,000 fibres of 7μm in diameter, the maximum pulling force $F_{pmax}$ that can be applied to the fibres is 9467.2N. The pulling force applied in correlation with $H_{f}$ for different values of fibre vertical displacement $\delta_{y}$ is plotted in Figure 9. A dashed horizontal line indicates the maximum pulling force of 9467.2N that can be applied to the fibres. For an $H_{f}$ value of 0.2mm, a minimum value of $\delta_{y}=0.39$ mm is required to not surpass the maximum pulling force. With increasing $H_{f}$, the required pulling force decreases until the value of $H_{f}=0.4$ mm. After this value, the pulling force increases at a constant slow rate with increasing $H_{f}$, for the same $\delta_{y}$. The proposed geometry should take into consideration the expected fibre displacement with a pulling force in an acceptable range for each value of $H_{f}$. The expected $H_{0}$ and $\delta_{y}$ values can be obtained by varying the height of the bottom wall peak at point B, adapting the pulling force accordingly.

## 5. Results Analysis

Seven numerical simulations were performed, considering the thickness of the fibres layer $H_{f}$$\left[\mathrm{mm}\right]$—0.2, 0.3, 0.4, 0.45, 0.475, 0.49 and 0.499, which correspond to the gap’s height $H_{0}$$\left[\mathrm{mm}\right]$—0.3, 0.2, 0.1, 0.05, 0.025, 0.01 and 0.001, respectively. These values were defined to obtain solutions from a high volume of polymer passing through the gap, for the cases where a significant part of the fluid is forced to cross the fibres.

### 5.1. Pressure and Velocity Analysis

**Figure 10:**
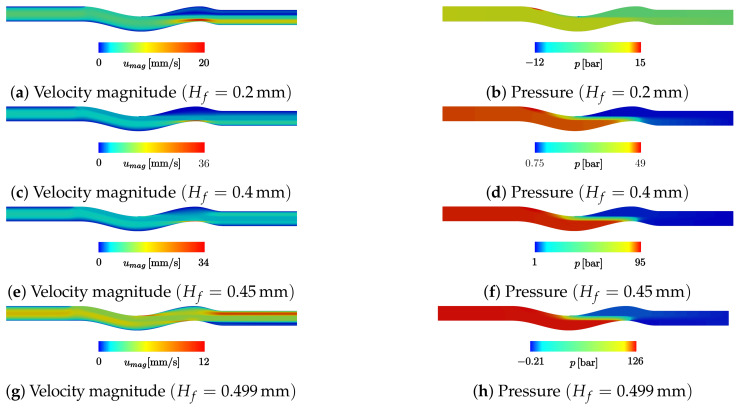
Velocity magnitude (**a**,**c**,**e**,**g**) and pressure map (**b**,**d**,**f**,**h**) for the performed simulations, with $H_{f}\;\left[\mathrm{mm}\right]$—0.2, 0.4, 0.45, 0.499, respectively.

The obtained solutions for the velocity magnitude and pressure map of four significant results are presented in Figure 10. For increasing values of $H_{f}$ and the corresponding decreasing $H_{0}$, as $H_{f}+H_{0}=0.5\;\mathrm{mm}$, the polymer flow through the fibres and the pressure drop both increase. With $H_{0}=0.3\;\mathrm{mm}$, the polymer mainly flows through the gap, imposing a small pressure drop (Figure 10a,b) along the polymer flow. In this case, the velocity profile in the die outlet is 0 for the section above the fibre, demonstrating that there is no fibre impregnation due to the small pressure drop between points A and B. With lower values of $H_{0}$, the pressure drop for the polymer to cross the gap increases (Figure 10d,f,h), and as $H_{f}$ increases at the same rate as $H_{0}$ decreases, the pressure needed for the polymer to cross the fibres decreases. This allows an increase in polymer volume flow, as can be observed by the outlet velocity profiles in Figure 10c,e,g. In the limit case of $H_{f}=0.499\;\mathrm{mm}$ ($H_{0}=0.001\;\mathrm{mm}$), almost no polymer flows through the gap, being completely forced to cross the fibres (Figure 10g) and imposing a pressure drop in excess of 126bar (Figure 10h), which cannot be met by the considered extruder.

At the fibres entry point to the extrusion die, just before the first wave, on the top wall (Figure 10b), there is a sudden increase in the source term due to the fibres’ movement with higher velocity than the polymer’s near the walls. In this study, either the fibres or their entrance were not considered; instead, a source term in the polymer momentum conservation equation was added to model the influence of the fibres on polymer flow. This local sudden increase in the momentum source causes the high-pressure values observed in that specific zone.

In the first wave, where the fibres detach the top wall, a sudden expansion and a consequent pressure drop can be observed. The expansion occurs due to the fibres functioning as a barrier to the polymer, blocking it from filling the space created between the fibres and the wall in the detachment point. Thus, the pressure in this region drops significantly and creates the pressure differential required for the fibre impregnation.

**Figure 11:**
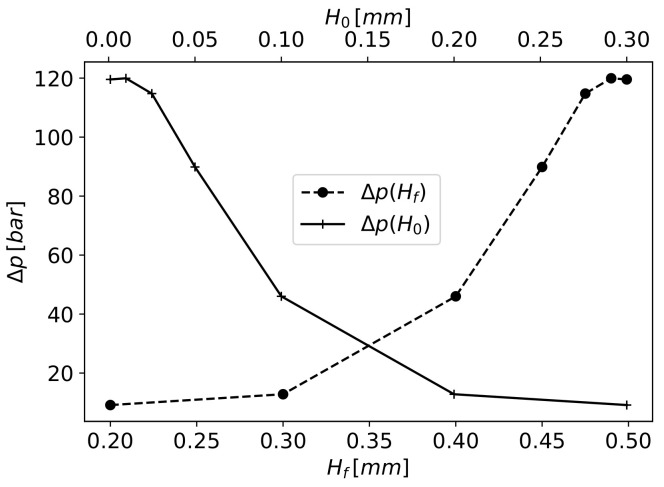
Pressure differential between the top and bottom sides of the fibres at the section from points A to B in relation to $H_{f}$ and $H_{0}$.

However, it can be observed that the main pressure drop occurs between points A and B for all the simulated cases. The evolution of this pressure drop in relation to $H_{f}$ and $H_{0}$ is presented in Figure 11. This evolution is not in accordance with the results presented in Figure 7 because, in the simulation case, the polymer volume forced through the fibres is not determined by the amount of polymer needed to fill the spaces but by the equilibrium between the pressure drop for the polymer to cross the gap $H_{0}$ and the pressure drop through the fibres.

### 5.2. Impregnation Quality

To evaluate the amount of polymer that crosses the fibres region, $q_{imp}$ was computed according to the following equation:

(20) $$q_{imp}=\int_{L_{In}+\frac{L_{1}}{2}}^{L_{Total}}max\left(-v\left(x,y=0\right),0\right)+max\left(v\left(x,y=-H_{f}\right),0\right)dx$$

**Figure 12:**
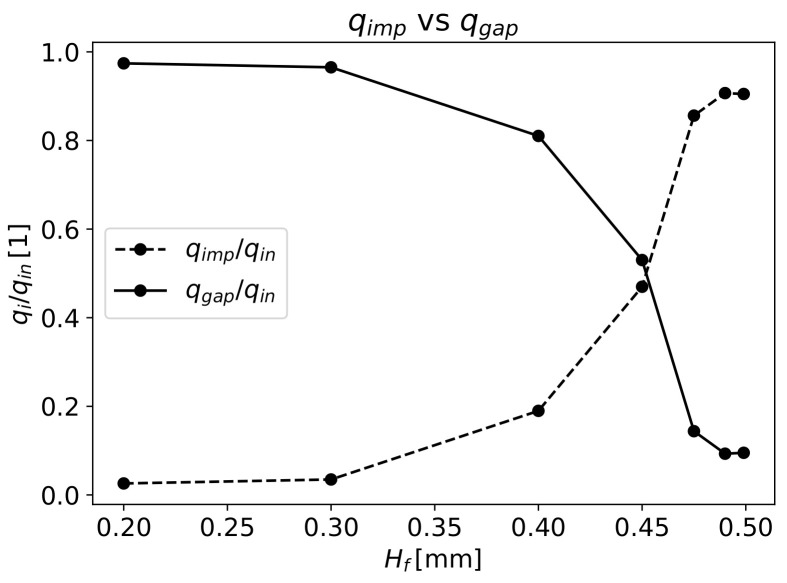
Polymer volume flow through the gap and through the fibres in relation to $H_{f}$.

The polymer volume flow through the fibres and through the gap in correlation with $H_{f}$ is observed in Figure 12. More than 95% of the polymer flows through the gap for the $H_{f}$ values of 0.2 and 0.3mm. The opposite occurs for $H_{f}$ values of 0.475, 0.49 and 0.499mm, where the polymer mainly flows through the fibres. Equilibrium is attained for the $H_{f}$ values of 0.4 and 0.45mm.

An appropriate impregnation is attained if the space in between fibres is fully filled by the polymer.

**Figure 13:**
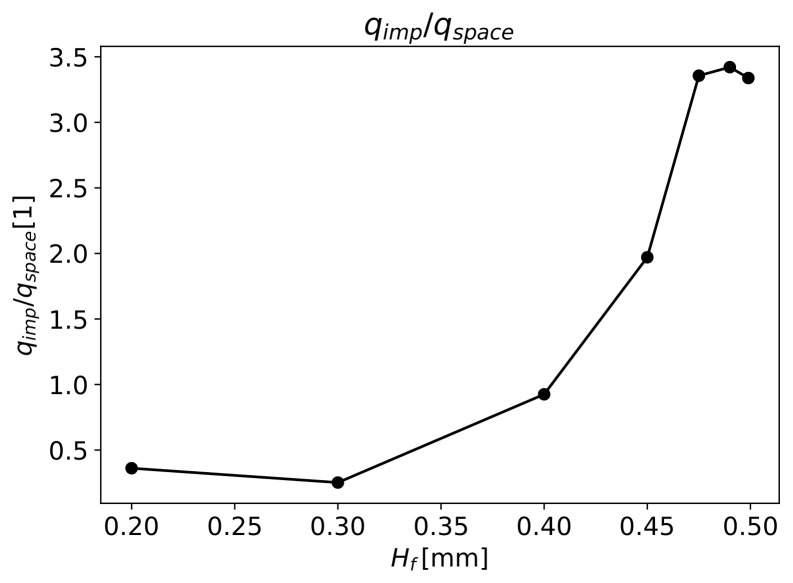
Impregnation fraction versus total fibres’ height.

Figure 13 presents the ratio between the polymer volume flow that enters into fibres $q_{imp}$ and the flow needed to fully impregnate the fibres $q_{space}$ in relation to $H_{f}$. It can be seen that when the fibres have less space between them, for $H_{f}=0.2\;\mathrm{mm}$, less than half of the required volume of fluid enters the objective fibres zone, resulting in poor impregnation. This occurs due to the low pressure needed for the majority of polymer to flow through the gap with $H_{0}=0.3\;\mathrm{mm}$. For this gap, the pressure drop through the fibres is 9bar. The same occurs for $H_{f}=0.3\;\mathrm{mm}$. Here, the pressure drop increases to 12.8bar, which allied to a lower $K_{⊥}$ with increasing $H_{f}$, slightly increases the volume flow through the fibres; however, since the $q_{space}$ in the fibre also increased, the ratio $q_{imp}/q_{space}$ decreased slightly, leading to lower fibre impregnation.

From the results of numerical simulations, the objective value of $q_{imp}/q_{space}=1$ is only achieved for $H_{f}$ higher than circa 0.4mm (see Figure 13) and a faster increase in this ratio is observed after that value. For $H_{f}=0.4\;\mathrm{mm}$, the pressure drop through the fibres is 46bar, which is similar to the obtained value with Darcy’s Law (Equation (14)), for the pressure drop $\left(52.6\;\mathrm{bar}\right)$ needed to fully impregnate the fibre with the same $H_{f}$.

At $H_{f}=0.45\;\mathrm{mm}$, the pressure drop through the fibres increases to 89bar, which is much higher than the pressure needed for $q_{imp}/q_{space}=1$ (51.6bar), in accordance with Darcy’s Law (Equation (14)). This behaviour is due to the low value of $H_{0}=0.05\;\mathrm{mm}$, substantially increasing the volume flow through the fibres and resulting in $q_{imp}/q_{space}\approx 1.97$. For the values of $H_{f}$$\left[\mathrm{mm}\right]$—0.475, 0.49 and 0.499, the pressure drop through the fibres converges to the value of 119bar and a $q_{imp}/q_{space}$ ratio of approximately 3. In this case, the volume flow through the gap is very low, stabilising the volume flow through the fibres, and the pressure drop needed to cross the fibres increases at a steady slow rate, leading to an almost constant pressure with increasing $H_{f}$.

### 5.3. Velocity in Fibres

**Figure 14:**
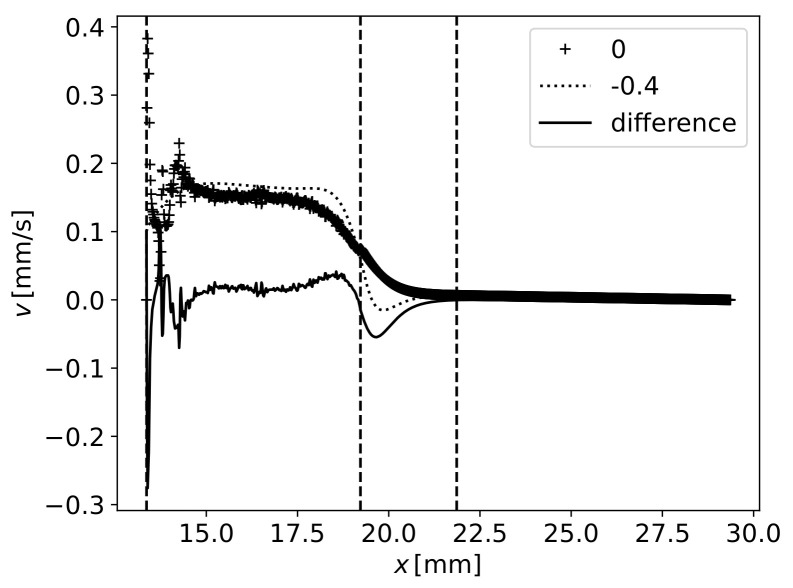
Vertical velocity component along the upper and lower limit of fibres with $H_{f}=0.4\;\mathrm{mm}$. Vertical lines at $x=L_{In}+L_{1}/2$, $x=L_{In}+L_{1}$ and $x=L_{In}+L_{1}+L_{2}/2$.

**Figure 15:**
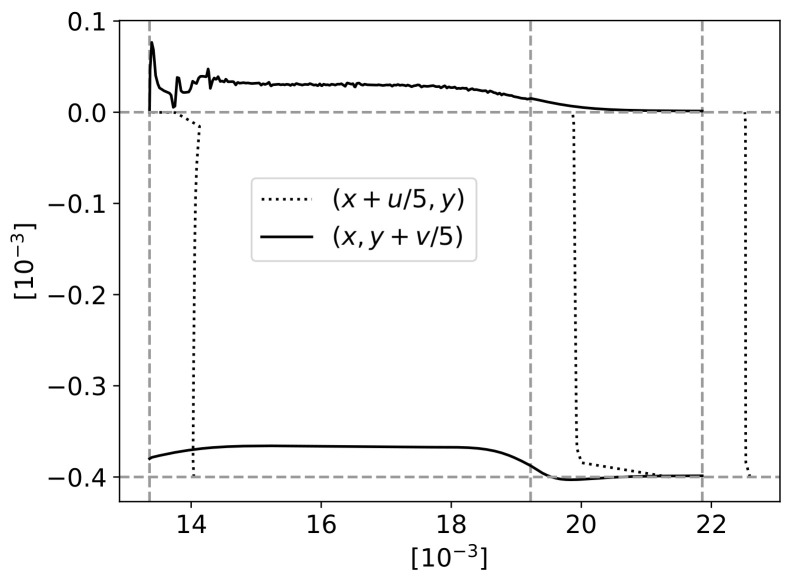
Velocity through horizontal section of the domain. Vertical lines at $x=L_{In}+L_{1}/2$, $x=L_{In}+L_{1}$ and $x=L_{In}+L_{1}+L_{2}/2$. Horizontal lines at $y=-H_{f}$ and y=0.

A more detailed analysis can be conducted on the polymer velocity along the fibres. Figure 14 presents the velocity vertical component along the upper limit $\left(y=0\;\mathrm{mm}\right)$ and lower limit $\left(y=-H_{f}\right)$ for the case of $H_{f}=0.4\;\mathrm{mm}$. The difference between these two values is also plotted. Vertical dashed lines were added at the fibres’ detach point from the top wall $\left(x=L_{In}+\frac{L_{1}}{2}\right)$ at the end of the first wave $\left(x=L_{In}+L_{1}\right)$ and the end of the second wave $\left(x=L_{In}+L_{1}+\frac{L_{2}}{2}\right)$ (Figure 3). A global upward movement of the fluid can be noticed up to the gap $\left(x=L_{In}+L_{1}\right)$, whereas after that, there is some downward movement. Regarding the difference between these two values, one of them can be positive (representing a source) up to the gap and negative (sink) afterwards. The mass conservation is assured by the variation of the horizontal component of velocity (Figure 15).

Figure 15 has the same reference vertical dashed lines as Figure 14 and horizontal lines at the fibres’ top $\left(y=0\;\mathrm{mm}\right)$ and bottom $\left(y=-H_{f}\right)$ limits. The velocity components were represented next to these lines, with a scale factor of 0.2, to better understand the global left-to-right movement as well as the difference that enables the mass conservation balanced with the vertical velocity component. After $x=L_{In}+L_{1}$ (point B), there is an expansion under the fibres’ region, leading to a pressure drop that induces a downward movement of the polymer.

### 5.4. Die Geometry Definition

Based on the numerical simulation results, die geometry can be proposed considering an $H_{f}$ range of probable values, since $H_{f}$ cannot be fully determined as it depends on the feeding system of the fibres, the pulling system at the exit of the die and the carbon fibre itself.

The new die design should allow a full impregnation of the fibres, corresponding to a $q_{imp}/q_{space}$ ratio equal or superior to 1, independently of $H_{f}$.

Considering the simplified model previously presented in the system of Equation (12), and solving the first equation to $H_{0}$, it is possible to define the gap’s height as a function of $H_{f}$ and Δp:

(21) $$H_{0}=\sqrt{L_{x}\frac{12\mu u}{\Delta p}}.$$

Imposing the pressure drop Δp to be equal to the pressure drop needed to fully impregnate the fibres, the maximum $H_{0}$ value was obtained to achieve the required pressure. The Δp needed to achieve a full impregnation of the fibres for a given $H_{f}$ can be obtained by Equation (14), as previously demonstrated in Section 4.2.

The polymer velocity *u* in the gap can be defined by

(22) $$u=\frac{q_{gap}}{wH_{0}},$$

where the polymer volume flow through the gap $q_{gap}$ is given by

(23) $$q_{gap}=u_{f}\left(H_{in}w-H_{f}w+N_{f}\frac{d_{f}^{2}}{4}\pi \right).$$

The simplified model can now be calibrated with the numerical simulation results, determining $L_{x}$ by

(24) $$L_{x}=-\frac{\Delta p_{x}H_{0}^{2}}{12\mu u}.$$

**Figure 16:**
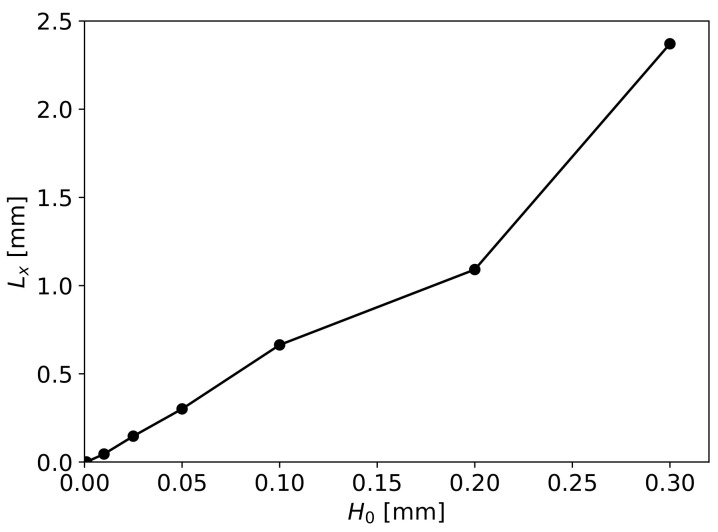
Obtained $L_{x}$ values in accordance with the numerical simulations results in function of $H_{0}$ and Equation (24).

With this equation and the obtained pressure drop values with numerical simulations, we obtain the $L_{x}$ values in the function of $H_{0}$. These results are presented in Figure 16, where a linear dependency can be identified allowing for the previously presented definition of $L_{x}=aH_{0}$. To evaluate the parameter *a*, the numerical simulation results with $H_{0}=0.001$ and 0.2 mm were excluded, since in these cases the pressure drop through the fibres and the gap is not equal, as it is implied in the definition of the simplified model. Thereby, the average value of *a* is 5.99 with a standard deviation of 0.42.

**Figure 17:**
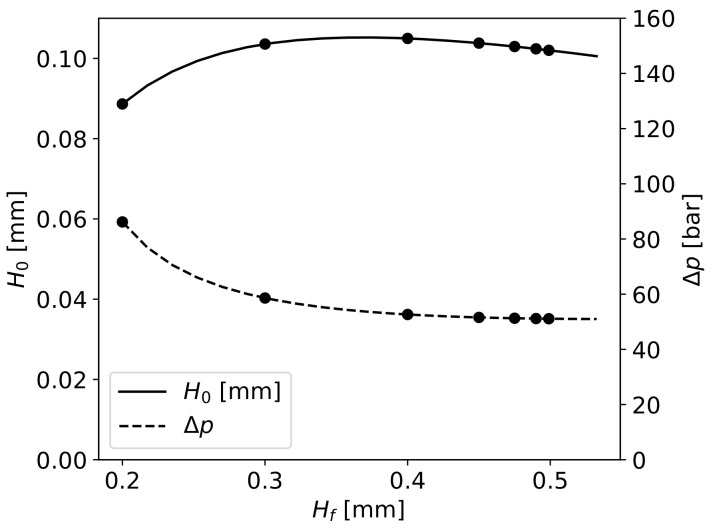
Obtained $H_{0}$ values and Δp required for full impregnation in function of $H_{f}$.

The calculated gap $H_{0}$ for $H_{f}$ varying from 0.15 to 0.5 mm (Equation (21)) is presented in Figure 17 as well as the required pressure drop Δp needed to fully impregnate the fibres. There is a close relation between the gap’s height, which assures the required pressure drop to fibres’ impregnation to a given $H_{f}$, and the required pressure drop itself.

**Figure 18:**
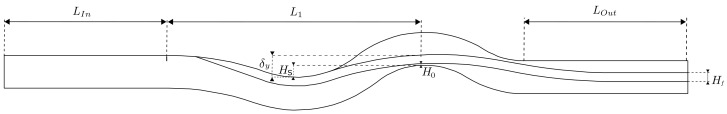
Die geometry considering the fibre displacement $\delta_{y}$.

The die geometry must be defined considering the required $H_{0}$ and the fibre displacement $\delta_{y}$ imposed by the pressure drop through the fibres. The die geometry considering the fibre deformation is shown in Figure 18. With the defined geometry, Equation (25) can be derived:

(25) $$\delta_{y}=H_{S}+H_{0}+H_{f},$$

where $H_{S}$ is the vertical overlap of the two die curves, as presented in Figure 18.

**Figure 19:**
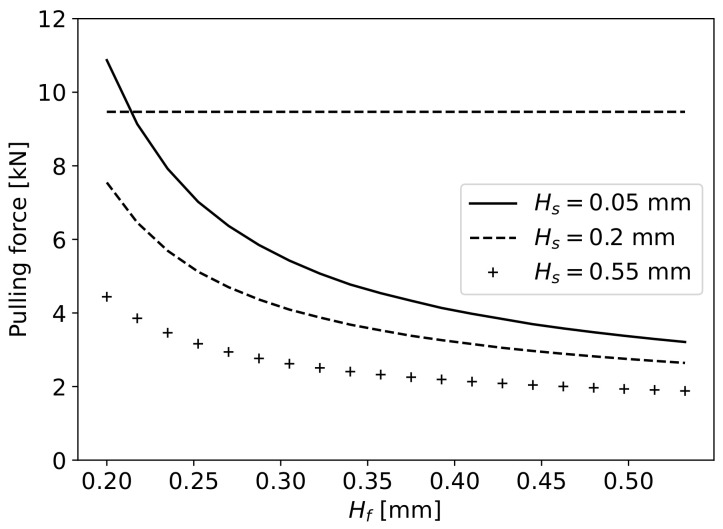
Pulling force in relation to $H_{f}$ for different values of $H_{S}$.

For a given value of $H_{S}$, the $\delta_{y}$ that the fibre should present for each $H_{f}$ to determine the required $H_{0}$ for full impregnation is obtained, and with $\delta_{y}$, the required pulling force $F_{p}$ can be calculated in relation to $H_{f}$ (Equation (17)). In Figure 19, the pulling force in relation to $H_{f}$ for different values of $H_{S}$ is presented. The $H_{S}$ value of 0.55mm was selected to allow an appropriate pulling force of fibres for any given $H_{f}$ value between 0.2 ($F_{p}=4441\;N$) and 0.5 mm ($F_{p}=1937\;N$). In the final proposed geometry, the values of $L_{in}$, $L_{1}$, $L_{2}$ and $L_{out}$ are kept constant, varying the maximum slope of the curves to achieve the desired value of $H_{s}$.

## 6. Conclusions

In this work, a simplified geometry of an extrusion die designed to impregnate carbon fibres with a polymeric matrix to produce CFRP tapes was analysed. During several numerical simulations, the thickness of the fibres layer was changed and, consequently, the gap that enables a shortcut to the polymer pass. The influence of the gap height was analysed, which showed that such a gap can lead to the occurrence of a shortcut that severely affects the impregnation conditions. The velocity of the polymer was analysed in more detail on the region where the majority of impregnation occurs, enabling us to understand the sections when the polymer crosses the fibres or travels parallel to them.

As gap height decreases, the pressure drop for the fluid to pass through the gap increases, as well as the pressure along the thickness of the fibres layer, leading to a greater volume of fluid crossing the fibres.

Finally, based on the simulation results, the gap height and pressure drop were defined in the function of fibre layer thickness to obtain a full impregnation of the fibres. These functions allowed the determination of a die geometry that imposes full impregnation of the fibres with an appropriate fibre-pulling force.

The defined die geometry can now be implemented for the experimental development of thermoplastic tape production by extrusion in the scope of the Carbo4Power project.

## Data Availability

Not applicable.

## Abbreviations

The following abbreviations are used in this manuscript:

AFP	Automated fibre placement
ATL	Automated tape laying
CFD	Computational Fluid Dynamics
EMI	Electromagnetic interference
FEA	Finite Element Analysis
FRP	Fibre-reinforced polymers
FVM	Finite Volume Method
SHM	Structural health monitoring
TP	Thermoplastic
TS	Thermoset
